# A prospective multicenter birth cohort in China: pregnancy health atlas

**DOI:** 10.1007/s10654-024-01157-x

**Published:** 2024-11-15

**Authors:** Si Zhou, Niya Zhou, Hanbo Zhang, Wenzhi Yang, Qingsong Liu, Lianshuai Zheng, Yuting Xiang, Dan Zheng, Yan Zhou, Siyi Wang, Danling Cheng, Jun He, Hong Wang, Wenbin Zhang, Liping Guan, Qiaoling Geng, Shihao Zhou, Hongbo Zhai, Hua Jin, Fei Hou, Shuzhen Wu, Jie Gao, Jing Yi, Luming Sun, Fengxiang Wei, Jianguo Zhang, Lei Yu, Xiao Yang, Leilei Wang, Lijian Zhao, Hongbo Qi

**Affiliations:** 1 Women’s Hospital, Zhejiang University School of Medicine, Hangzhou, 310006 Zhejiang China; 2 Chongqing Research Center for Prevention & Control of Maternal and Child Diseases and Public Health, Women and Children’s Hospital of Chongqing Medical University, Chongqing, 401147 China; 3 BGI Genomics Co., Ltd., Shenzhen, 518083 China; 4 Hebei Maternal and Child Genes and Health Industry Technology Research Institute, Shijiazhuang, 050000 China; 5 Shijiazhuang BGI Medical Laboratory Co., Ltd., Shijiazhuang, 050000 China; 6 Prenatal Diagnosis Department, Chengdu Women’s and Children’s Central Hospital, Chengdu, 611731 China; 7 Lianyungang Maternal and Child Health Hospital, Lianyungang, 222000 China; 8 Department of Obstetrics, Dongguan People’s Hospital, Dongguan, 523059 China; 9 Dongguan Key Laboratory of Major Diseases in Obstetrics and Gynecology, Dongguan, 523059 China; 10 Guiyang Maternal and Child Health Care Hospital, Guiyang, 550000 China; 11 Inner Mongolia Maternity and Child Health Care Hospital, Hohhot, China; 12 Inner Mongolia Autonomous Region Engineering Research Center for Medical Genetics, Hohhot, China; 13 College of Life Sciences, University of Chinese Academy of Sciences, Beijing, 100049 China; 14 Longgang Maternal and Child Health Hospital (Longgang Maternal and Child Clinical College of Shantou University Medical College), Shenzhen, 518100 China; 15 Hunan Provincial Key Laboratory of Regional Hereditary Birth Defects Prevention and Control, Changsha Hospital for Maternal & Child Health Care Affiliated to Hunan Normal University, Changsha, China; 16 Hebei Province Key Laboratory of Environment and Human Health, Department of Epidemiology and Statistics, School of Public Health, Hebei Medical University, Shijiazhuang, China; 17 Department of Obstetrics, School Of Medicine, Affiliated Hangzhou First People’s Hospital, Westlake University, Hangzhou, China; 18 Department of Prenatal Diagnosis, Jinan Maternal and Child Health Hospital, JinanShandong Province, 250001 China; 19 Department of Medical Administration, Dalian Women and Children’s Medical Group, DaLian, 116033 China; 20 Shanghai First Maternity and Infant Health Hospital, Shanghai, 201204 China; 21 Department of Fetal Medicine & Prenatal Diagnosis Center, Obstetrics and Gynecology Hospital Affiliated to Tongji University, Shanghai, 201204 China; 22 Department of Obstetrics, Chengdu Women’s and Children’s Central Hospital, Chengdu, 611731 China; 23 Medical Technology College of Hebei Medical University, Shijiazhuang, 050017 China; 24 Department of Obstetrics and Gynecology, Women and Children’s Hospital of Chongqing Medical University, Chongqing, 401147 China

**Keywords:** Birth cohort, Prospective study, Multi-center study, Multi-omics, DOHaD

## Abstract

The China Prospective Multi-Center Birth Cohort Study was launched in 2022. In collaboration with medical centers in 12 cities, it aims to establish a high-quality, multidimensional cohort comprising 20,000 natural pregnancy and assisted reproductive families. As of June 26, 2024, 12,911 pregnant women have participated in this study, and 161,122 biological samples have been collected. These samples cover four critical periods (early pregnancy, mid-pregnancy, late pregnancy, and postpartum) and comprise 10 different types such as serum, plasma, and urine. The study has collected comprehensive information from early pregnancy to newborns. The participants have an average age of 29.76 years, an average height of 160.46 cm, an average pre-pregnancy BMI of 23.11, and an average BMI of 27.25 before delivery. The cohort includes individuals from 26 ethnic groups, with 25 minority groups comprising 5.03% of the population. Guizhou Province exhibits the highest percentage of ethnic minorities at 24.96% and Guangdong Province owns the highest proportion (12.22%) of women with two or more children. The prevalence of thalassemia in Guangdong Province is seven times higher than in other provinces. Among pregnant women over 35 years old, the prevalence of gestational diabetes mellitus is twice that of women under 35. Additionally, the prevalence of preeclampsia in women with assisted reproductive pregnancies is more than twice that of those with natural pregnancies. The study extensively collected diverse data and biological samples, making this cohort an ideal candidate for DOHaD field and multi-omics research.

**Supplementary Information:**

The online version contains supplementary material available at 10.1007/s10654-024-01157-x.

## Introduction

Maternal health is a global topic drawing significant attention, especially in developing countries, where maternal and child health issues are particularly prominent [1–3]. The average age of pregnant women is gradually increasing around the world [4] and the incidence of pregnancy complications is also rising [5, 6]. Studies have shown that pregnancy complications [7] have a profound impact on maternal and child health [8–11]. Previous studies revealed that age [5], ethnicity [12], assisted reproductive technology (ART) [13, 14] and family history [15, 16] are important causes of gestational diseases, such as serious complications including preeclampsia (PE) and premature birth. A bunch of cohorts have been established addressing adverse pregnancy outcomes [17], maternal and neonatal/children health [18, 19].

As a country with vast territory holding diverse geographic features and a blend of multiple ethnicities, China exhibits a high degree of diversity in prenatal health issues [20]. The relaxation of birth policies in China [21] and the postponement of childbearing due to life stress lead to an increased average maternal age among pregnant women. Therefore, it is imperative to establish a multi-center cohort study in China that reflects the polymorphic landscape of prenatal health.

Facing this challenge, we aim to build a high-quality, multi-center cohort that includes 20,000 families from 12 cities in China. The prospective multi-center birth cohort collects blood, urine, and vaginal discharge from pregnant women during early, middle, late pregnancy and delivery. Umbilical cord blood, amniotic fluid, placenta, and meconium samples are also collected at delivery, providing a comprehensive biological samples resource allowing for further investigation.

In recent years, the advancement of omics technologies including genomics [22], transcriptomics [23], proteomics [24], metabolomics [25] have rapidly developed and shown great potential in disease prediction and prevention. However, carrying out multi-omics research calls for delicate processing and storage of high-quality samples, which poses a great challenge to cohort construction, especially multi-centered ones. The prospective multi-center birth cohort of this study collected high-quality biological samples and reliable clinical information, thus enabling multi-omics big data studies covering the entire perinatal period.

In this study, we collected comprehensive information of pregnant women and newborns, including demographic characteristics, parity, BMI, conception method, birth weight, Apgar score and other information of newborns. Due to the data granularity and size, this baseline information provides us with comprehensive initial data to support subsequent multi-omics analyses and longitudinal follow-up studies, making our cohort a potential catalyst for further developmental origins of health and disease research and improving maternal and child health in China and globally.

### General and specific objectives

**Fig. 1:**
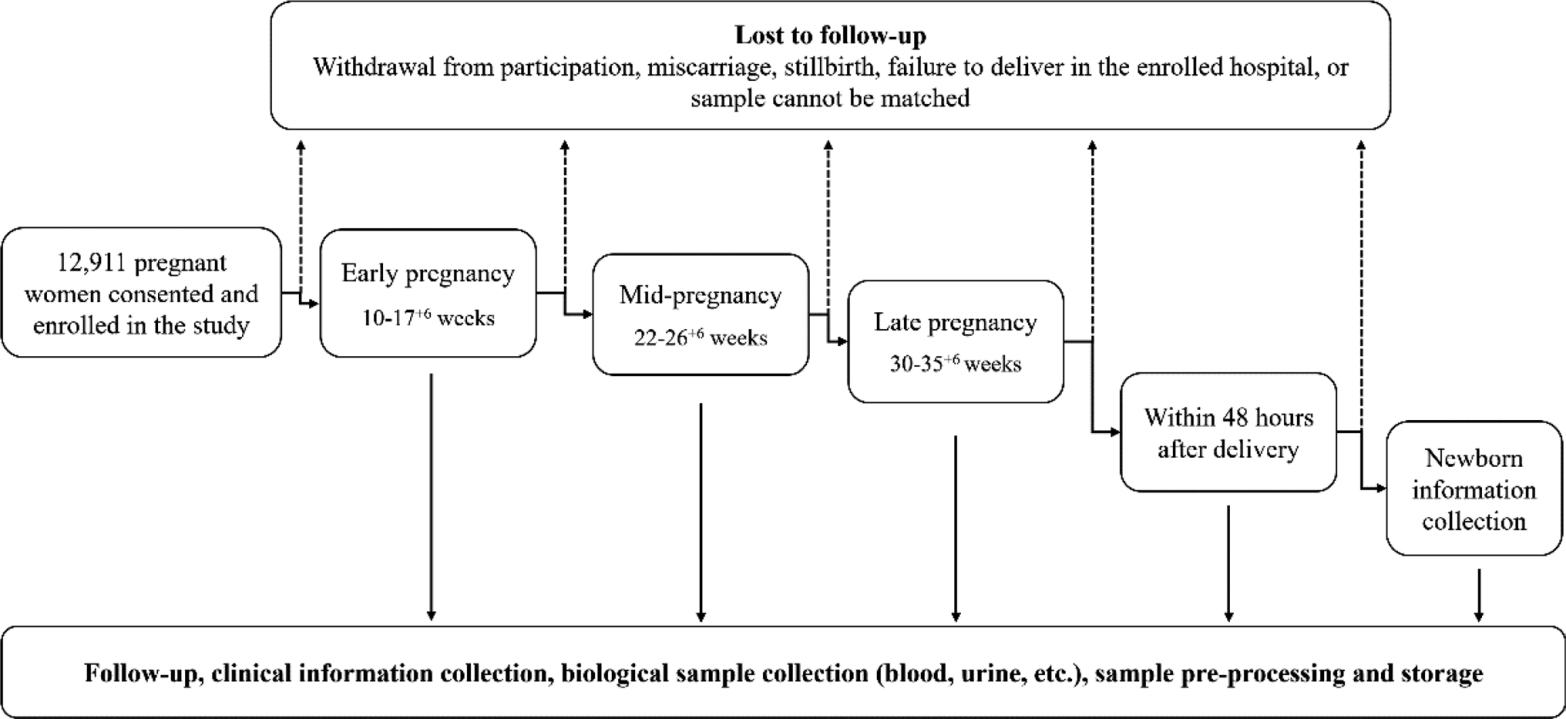
Flow chart of participant enrollment, sample collection, and follow-up in the prospective multicenter cohort

With the rapid development of artificial intelligence and the rise of multi-omics big data research, maternal and child health studies, particularly cohort studies, face higher demands. To address this, the China Prospective Multi-Center Birth Cohort Study was launched in 2022, uniting medical centers in 12 cities and aiming to establish a high-quality, multidimensional cohort comprising 20,000 natural pregnancy and assisted reproductive families. The cohort features extensive geographical coverage, a large survey population, diverse biological samples, longitudinal design and multi-omics integration. By collecting biological samples and comprehensive data at different perinatal stages (Fig. 1), it is possible to systematically and multidimensionally study maternal and child multi-omics big data, providing valuable insights into the developmental origins of health and disease, particularly within the context of Chinese populations.

Our specific objectives include but are not limited to:

Reproductive health and pregnancy disease research: Collect baseline information from reproductive-age couples to explore potential risk factors for reproductive health.

Maternal and child health and development research: Analyze the impact of assisted reproductive technologies on pregnancy outcomes and offspring health, and study the influence of genetic and environmental risk factors on maternal and child health.

Advancing multi-omics research: Promote the integration and analysis of multi-omics data through genomics, transcriptomics, proteomics, and metabolomics research, driving scientific research and technological innovation in the field of maternal and child health.

Disease prediction and personalized treatment: Utilize multi-omics data to develop risk prediction models for pregnancy diseases and promote the application of personalized treatment methods.

Accumulation of large-scale multi-modal data: Support the development and refinement of precision medicine large language models through the accumulation of large-scale, multi-modal data.

## Methods

### Study design and population

**Fig. 2:**
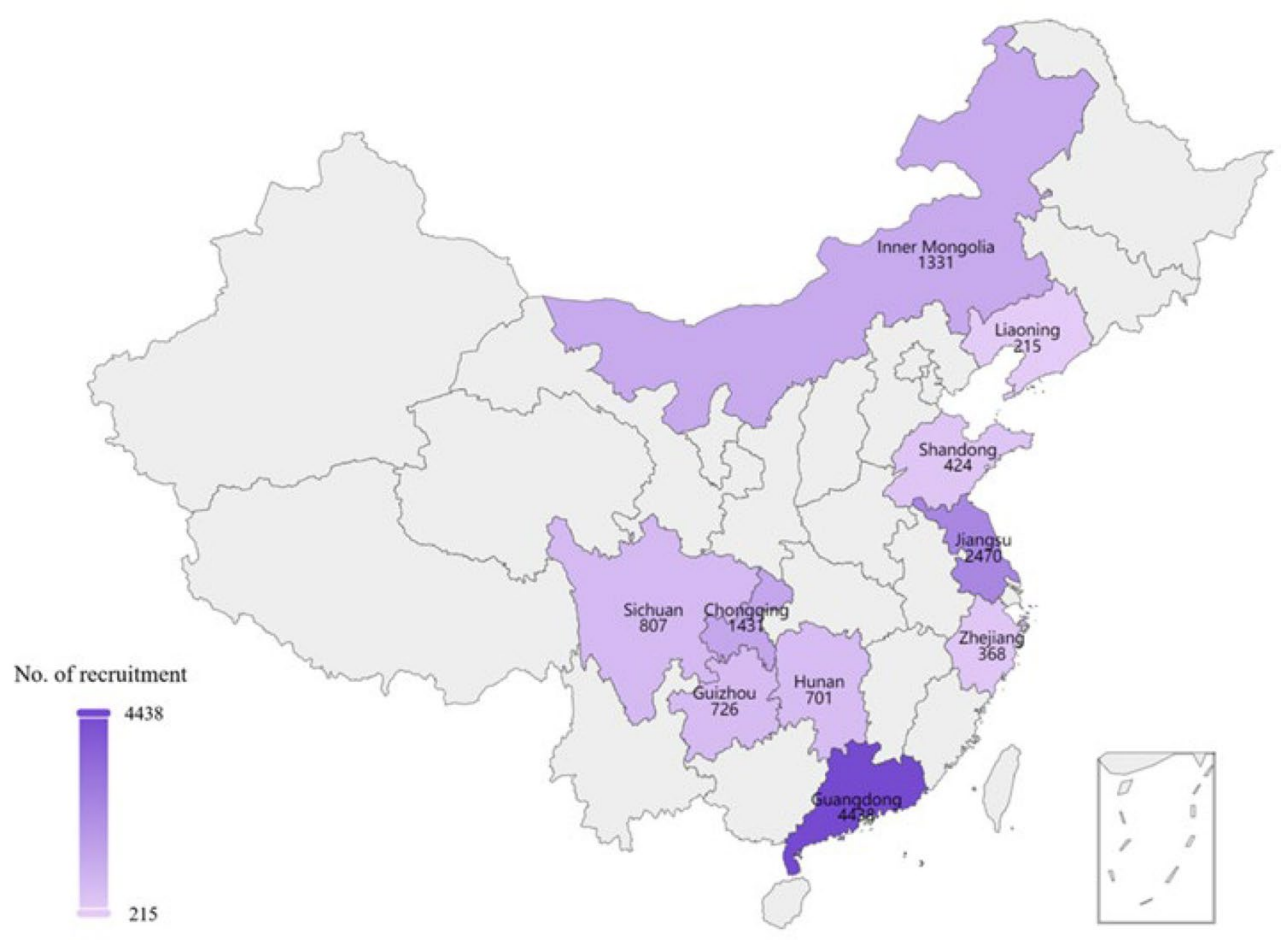
Geographical distribution of the cohort enrolled in this study. The study contains 10 provinces, cities, autonomous regions in China

This study is an ongoing prospective multi-center cohort study, designed in 2021 and commenced with the enrollment of pregnant women on November 25, 2022. The study is participated by 12 medical centers, covering 12 cities and 10 provinces or autonomous regions in China (Fig. 2), and aims to establish a high-quality, multidimensional cohort comprising 20,000 natural pregnancy and assisted reproductive families. From 10 to 17^+6^ weeks of gestation, blood and urine samples are collected from the pregnant women. During the periods of 22 to 26^+6^ weeks and 30 to 35^+6^ weeks of gestation, blood, urine, and cervicovaginal fluid (CVF) samples are collected. Within 48 h postpartum, samples including maternal blood, umbilical cord blood, placenta, amniotic fluid, and meconium are collected. Additionally, pregnant women are required to complete questionnaires at the time of enrollment and during each biological sample collection period (Fig. 1).

The inclusion criteria for this study are as follows:

1. Signing an informed consent form and agreeing to participate in the study, being a resident in the local area for more than six months;
2. No communication barriers;
3. Planning to undergo routine prenatal examinations and delivery at the enrolling hospital, and aged between 18 and 45 years old.
4. The exclusion criteria include pregnant women who are unable to understand the study content or unable to sign the informed consent form.

## Data collection

### Baseline information

The data for this study are obtained through the following methods (Table 1): (1) Information collected from pregnant women through questionnaires, including age, ethnicity, last menstrual period, height, weight, and medical history; (2) Data recorded by professionally trained doctors through face-to-face interviews, covering health status, gravidity and parity, place of residence, lifestyle, and reproductive status; (3) Perinatal information of pregnant women was obtained through physical measurements, laboratory tests, telephone follow-up and hospital electronic medical record system; (4) Biological sample information collected through the prospective multi-center birth cohort study.

**Table 1:** Biological samples, data, and collection methods in the prospective multicenter cohort

Stages	Biological samples	Data	Collection method
Early pregnancy 10–17^ +6^ weeks	Blood, urine	Demographic characteristics: age, ethnicity, height, weight, place of residence, education level, place of residence Health status: Blood pressure, BMI, other diagnoses from clinical doctors Lifestyle: Smoking history, drinking history; Reproductive status: Last menstrual period, gravidity and parity, gestational week, mode of conception, singleton or multiple pregnancy	Questionnaire, face-to-face collection by doctors
Mid-pregnancy 22–26 ^+6 ^weeks	Blood, urine, cervicovaginal fluid	Height, weight, blood pressure, gestational week of sampling	Questionnaire
Late pregnancy 30–35 ^+6^ weeks	Blood, urine, cervicovaginal fluid	Height, weight, blood pressure, gestational week of sampling	Questionnaire
Within 48 h after delivery	Blood, umbilical cord blood, placenta, amniotic fluid	Delivery method, gestational week of delivery, delivery outcome, newborn gender, birth height and length, Apgar score, pregnancy complications	Hospital electronic medical record system, telephone follow-up

### Clinical information and diagnosis

Preterm birth is labor occurring between 28 and 37 weeks gestation [26]. Fetal growth retardation (FGR) is defined as an ultrasound-estimated fetal weight or abdominal circumference less than the 10th percentile for gestational age [27]. Gestational diabetes mellitus (GDM) refers to diabetes diagnosed via OGTT during 24–28 weeks of pregnancy that is not clearly overt diabetes [28]. Macrosomia is defined as growth beyond 4,000 g [29]. Gestational hypertension [30, 31] is defined as blood pressure greater than or equal to 140mmHg systolic or 90mmHg diastolic after 20 weeks of pregnancy when previous blood pressure was normal. PE is mainly defined as the new onset of hypertension and proteinuria or a protein/creatinine ratio greater than or equal to 0.3. PE with severe features is defined when features including blood pressure over 160/110 mmHg, headache, vision disturbance, elevated AST/ALT, etc. are presented. The diagnosis of intrahepatic cholestasis of pregnancy (ICP) is via the presence of pruritus in the third trimester with maternal total serum bile acids level over 10  μmol/L and excluding other diagnosis [32],in severe ICP, the cut off is 40 μmol/L [33]. Premature rupture of membrane (PROM) is the rupture of membranes before the onset of labor [34]. Hypothyroidism during pregnancy is defined as TSH level above 4.0 mU/L [35]. Diagnosis of uterine leiomyomas is generally presented as an enlarged uterus that is often irregular in shape during physical examination [36].

### Followed up strategies

For pregnant women who consent to participate (Fig. 1), the first sampling occurs in early pregnancy, between 10 and 17^+6^ weeks of gestation. Blood samples are collected by healthcare personnel, while urine samples are self-collected by the participants. During this visit, participants also complete a questionnaire, and professionally trained physicians collect and record information through face-to-face interviews.

The second and third sampling and follow-up visits occur during the second trimester (22–26^+6^ weeks) and the third trimester (30–35^+6^ weeks), respectively. Blood and CVF samples are collected by healthcare personnel, urine samples are self-collected, and questionnaires are completed by the participants. Doctors collect and recorded information through face-to-face interviews during routine prenatal examinations.

The fourth sampling and follow-up visits occur within 48 h post-delivery. Healthcare personnel collect maternal blood, umbilical cord blood, placenta, amniotic fluid, and meconium. Doctors collect and record information about maternal and newborn. Comprehensive information about the pregnant women and the collection of biological samples was collected at all four stages (Table 1).

### Biological sample collection

Blood sample collection: Blood samples are collected using tubes containing EDTA-K2 anticoagulant and serum-separating tubes for obtaining plasma and serum samples, respectively. EDTA-K2 tubes are centrifuged at 1600 g for 10 min at 4 °C to separate the supernatant, followed by a second centrifugation at 16000 g for 10 min. Serum-separating tubes are centrifuged at 1600 g for 10 min. Post-centrifugation, plasma, serum, and buffy coat samples are aliquoted into separate tubes and stored at − 80 °C.

Urine sample collection: Pregnant women collect approximately 20 mL of midstream urine using a disposable urine cup. The urine is then transferred into two 15 mL tubes, securely capped, and handed over to the medical staff for storage at − 80 °C.

CVF sample collection: Obstetricians collect secretions using a speculum from the posterior fornix of the vagina or from the lateral walls at one-third to one-half of the vaginal canal. The swabs are placed in dry, sterile cryotubes and stored at − 80 °C.

Amniotic fluid sample collection: Obstetricians collect amniotic fluid in 15 mL sterile centrifuge tubes. The samples are centrifuged at 1600 g for 10 min at 4 °C. The supernatant and cell pellet are then separated into cryotubes and stored at − 80 °C.

Placental tissue sample collection: Placental tissue samples are collected from the maternal and fetal sides of the placenta. The collected tissue samples are rinsed with saline and stored at − 80 °C.

By adhering to these standardized protocols, we ensure the integrity and quality of the biological samples for subsequent multi-omics analyses.

### Quality control

This study has established strict standardized procedures for the collection, processing, transportation and storage of samples. Blood samples are centrifuged within 4 h after collection, and the volume, properties, temporary storage conditions and processing time of the samples are recorded. We utilize sterile, nuclease-free, and low chemical leaching sample storage tubes (AXYGEN, MCT-200-C) and standard cryogenic storage boxes to ensure the quality and accurate positioning of the samples. During transportation, samples are transferred using cold chain logistics, with sufficient dry ice to maintain a low-temperature environment, and temperature monitoring and recording devices are used to ensure that the temperature during transportation remains at − 80 °C. After verification and cross-checking, all samples are stored in freezers at − 80 °C, which are equipped with low-temperature recorders to continuously monitor the storage temperature.

### Ethical considerations

All participants signed informed consent, including the collection of biological samples and clinical information at different stages of pregnancy, and multi-omics testing and analysis based on these samples. We take all necessary measures to ensure the confidentiality of personal information. This study was approved by the Ethics Committee of Women and Children’s Hospital of Chongqing Medical University (Approval No. 2022–038) and by the Ministry of Science and Technology of the People’s Republic of China (Approval No. 2023-CJ0981).

### Statistical analysis

In this study, a comprehensive statistical analysis of the baseline data was conducted to provide a detailed description of the baseline characteristics of the enrolled population. For each continuous variable, we calculated the mean, standard deviation, and 95% confidence interval to describe the central tendency and dispersion of the data. These statistics provided us with the basic distribution characteristics of each variable. To compare whether there were significant differences between different groups, we used the chi-square test and Fisher’s exact test. These methods helped us accurately assess the significance of differences between groups.

## Result

### Baseline characteristics of the participants

This prospective multi-center birth cohort study has a rapid increase in enrollment since its launch in November 2022 (Supplement Fig. 1). As of June 26, 2024, a total of 12,119 pregnant women have consented to participate, with baseline characteristics detailed in Table 2. The average age of the pregnant women is 29.76 years old and 8.21% are over 35 years old. The average height of the participants is 160.46 cm. The study includes 26 ethnic groups, with 5.03% from 25 minority groups (Supplement Fig. 2). Over 90% of the participants reside in urban areas and the average pre-pregnancy BMI is 23.11. Natural pregnancies account for 95.4%, and 98.75% are singleton pregnancies. 60.24% of pregnant women delivered vaginally, the average gestational age was 38.70 weeks, and the average BMI before delivery is 27.25. 52.94% of the newborns are male, with an average weight of 3186.90 g and an average height of 49.60cm. More than 70% of the newborns have a length of 50 cm or greater.

**Table 2:** Baseline characteristics of participants in the prospective multicenter cohort

Characteristics	No. of samples (Mean, 95%CI/median)	Percentage
Maternal ethnicity		
Han	10,147	94.97%
Minority	537	5.03%
Age		
Average age	29.76, 29.68–29.83	
≤ 26	2578	20.51%
27–30	4983	39.64%
31–35	3979	31.65%
> 35 years old	1032	8.21%
Average height	160.46, 157.76–163.16	
< 160 cm	4774	41.08%
160–165 cm	4043	34.79%
≥ 165 cm	2804	24.13%
Average BMI before pregnancy	23.11, 23.02–23.19	
< 18.5	956	8.24%
18.5–25	7850	67.70%
25–30	2143	18.48%
≥ 30	646	5.57%
Average BMI before delivery	27.25, 27.13–27.37	
< 18.5	34	0.83%
18.5–25	1134	27.62%
25–30	2028	49.39%
≥ 30	910	22.16%
Pregnancy method		
Natural pregnancy	10,406	95.40%
Assisted pregnancy	502	4.60%
Single	4729	98.75%
Twin	60	1.25%
Abortion, induced abortion, Stillbirth	34	0.70%
Primipara	5620	63.26%
Pregnant woman with ≥ 2 Parities	708	7.97%
Smoking history	20	0.33%
Drinking history	89	1.42%
Permanent residence	5647	
Rural	537	9.51%
Urban	5110	90.49%
Education level	4059	
Below undergraduate	1070	26.36%
Undergraduate	2784	68.59%
Master’s degree	198	4.88%
PhD	7	0.17%
Delivery method		
Natural delivery	2454	60.24%
Cesarean section	1620	39.76%
Average gestational age	38.70, 38.46–38.94	
< 37 weeks	334	6.97%
≥ 37 weeks	4455	93.03%
Average birth weight	3186.90, 3147.90–3225.90	
Gender of newborn	4452	
Male	2357	52.94%
Female	2095	47.06%
Average height of newborn	49.60, 49.54–49.65	
< 45 cm	77	1.81%
45–50 cm	1154	27.08%
≥ 50 cm	3031	71.12%

Given the multi-center nature of this study, we analyzed the distribution of ethnicity, age, height, parity, and education level across different provinces (Table 3). Guizhou Province, known for its ethnic diversity, has the highest proportion of minority among the 12 cities with 24.96%. Pregnant women in Liaoning Province have the highest average height at 164.21 cm, followed by those in Inner Mongolia Autonomous Region (163.32 cm) and Shandong Province (162.97 cm), this may be related to geographic factors. In terms of parity, 63.26% of the pregnant women are primiparous. Additionally, Shandong Province has the highest proportion of pregnant women over 35 years old (16.75%). Guangdong province (12.22%) has the highest percentages of pregnant women with two or more children, which may be influenced by urban living pressures and local customs. Regarding educational levels, the majority of pregnant women hold a bachelor’s degree (68.59%) and the distribution of education levels varies across provinces, likely reflecting the unique urban characteristics of each province (Table 3). These diverse and detailed baseline characteristics provide a solid foundation for further multi-omics analysis and longitudinal follow-up studies.

**Table 3:** Baseline information for different regions

	Guangdong Province	Guizhou Province	Chongqing City	Jiangsu Province	Liaoning Province
Percentage of ethnic minorities	3.76%	24.96%	4.01%	0.87%	2.31%
Average age	29.67	29.72	29.80	28.22	31.66
Percentage over 35 years old	12.34%	10.10%	7.12%	0.33%	13.93%
Height (average)	158.43	158.58	159.40	162.84	164.21
Percentage of first-time mothers	49.30%	79.55%	72.33%	66.07%	79.77%
Parity ≥ 2	12.22%	4.51%	2.39%	5.58%	3.47%
Undergraduate or below	38.46%	29.25%	10.53%	–	–
Undergraduate	58.70%	63.68%	88.37%	–	–
Master and PhD	2.84%	7.08%	1.11%	–	–

	Inner Mongolia Autonomous Region	Hunan Province	Shandong Province	Zhejiang Province	Sichuan Province
Percentage of ethnic minorities	12.93%	4.95%	1.90%	2.10%	3.73%
Average age	29.65	30.34	30.47	30.16	29.97
Percentage over 35 years old	2.34%	12.58%	16.75%	7.51%	9.24%
Height (average)	163.32	160.33	162.97	160.87	159.72
Percentage of first-time mothers	77.82%	70.75%	51.53%	82.28%	69.37%
Parity ≥ 2	2.51%	3.77%	8.64%	2.10%	2.76%
Undergraduate or below	10.73%	33.33%	36.43%	–	13.61%
Undergraduate	81.06%	66.67%	57.36%	–	78.12%
Master and PhD	8.21%	–	6.20%	–	8.27%

### Statistics of clinical complications

In light of the large population included and medical records extensively retrieved, this cohort brings us abundant clinical information, including common obstetric and gynecologic complications, neonatal disease, hepatobiliary disease, infectious disease, endocrine disease, metabolic disease and genetic disease (Table 4). Among 4865 pregnant women who have given birth, several common diseases are observed: premature rupture of membrane (18.29%), GDM (16.74%), hypothyroidism (8.44%), thalassemia (5.70%), fetal distress (5.16%), preterm birth (4.92%), uterine leiomyoma (4.36%), macrosomia (3.32%), PE (2.74%), FGR (1.78%), ICP (1.54%) and Polycystic ovary syndrome (PCOS) (0.57%).

**Table 4:** Prevalence of common pregnancy complications

Disease	No	%	Disease	No	%
premature rupture of membranes	953	18.29%	FGR	95	1.78%
GDM	847	16.74%	ICP	64	1.54%
hypothyroidism	417	8.44%	chronic hypertension	52	1.00%
thalassemia	335	5.70%	PE with severe feature	44	0.95%
fetal distress	281	5.16%	G6PD	69	0.94%
asymptomatic streptococcal infection	349	5.15%	thyroiditis	35	0.74%
preterm birth	276	4.92%	gestational heart disease	61	0.67%
uterine leiomyoma	193	4.36%	PCOS	34	0.57%
macrosomia	171	3.32%	hyperthyroidism	32	0.56%
gestational hypertension	142	2.96%	fetal heart abnormalities	40	0.40%
hyperuricemia	225	2.93%	hypercholanaemia of pregnancy,	19	0.24%
PE	126	2.74%	severe ICP	11	0.22%

*ICP intrahepatic cholestasis of pregnancy, *FGR* fetal growth restriction, *GDM* gestational diabetes mellitus *PCOS* polycystic ovary syndrome, *PE* preeclampsia, *PROM* premature rupture of membranes

Mode of conception and advanced maternal age has been associated with adverse pregnancy outcomes. We analyzed the disease prevalence in women receiving ART and women with advanced maternal age (Supplement Tables 1 and 2). The subfertility of pregnant women receiving ART likely increased their vulnerability in face of gestational stress and lead to higher risk of adverse outcomes. We found that compared to women receiving natural pregnancy, women receiving ART exhibited a significantly higher prevalence of several disease including preterm birth(*P* = 0.0002), GDM (*P* = 0.046), PE (*P* = 0.002), PCOS (*P* = 0.048). Additionally, we found that compared to women at age 35 and older, women younger than 35 showed a significantly lower prevalence in preterm birth (*P* = 0.0042) and GDM (*P* < 2.2e^−16^). However, there is no significant difference between two groups in FGR, PE, ICP, macrosomia and fetal heart abnormalities.

Thalassemia is a kind of hereditary hemolytic blood disease, which is mainly prevalent in tropical and subtropical regions of the world [37]. In the subtropical Guangdong province, 266 cases were found among 1,503 women, while in other regions only 79 cases were found among 3,339 women. Additionally, we also observed 9 fetal chromosome abnormalities and 4 participants with fetal genetic abnormalities in the cohort (Supplement Table 3), this might be de novo mutation happened during gestation.

### Collection and analysis of biological samples

As of June 26, 2024, this study has collected a total of 161,122 biological samples (Table 5), covering four key periods: early pregnancy, mid-pregnancy, late pregnancy, and postpartum. These samples include serum, plasma, urine, buffy coat, cervicovaginal fluid (CVF), amniotic fluid, placenta, umbilical cord plasma and buffy coat, providing valuable resources for comprehensive, multidimensional maternal and child health research. In the early pregnancy period, we collected samples from 8,743 pregnant women, including 62,184 blood samples with an average gestational age of 13^+5^ weeks and 23,368 urine samples with an average gestational age of 14^+3^ weeks. During mid-pregnancy, we collected samples from 4,383 pregnant women, consisting of 32,933 blood samples with an average gestational age of 24^+4^ weeks, 10,838 urine samples with an average gestational age of 23^+6^ weeks, and 1,683 CVF samples with an average gestational age of 28.5 weeks. In the late pregnancy period, we collected samples from 2,416 pregnant women, including 15,888 blood samples with an average gestational age of 34 weeks, 2,257 urine samples with an average gestational age of 32^+4^ weeks, and 2,192 CVF samples with an average gestational age of 34^+1^ weeks. Additionally, postpartum samples were collected from 670 women, comprising a total of 9,779 samples. The collection of these diverse samples spans from early pregnancy to postpartum, providing comprehensive biological data across different stages of pregnancy.

**Table 5:** Information on samples collected in the prospective multicenter cohort

Sample type	Early pregnancy			Mid-pregnancy			Late pregnancy			Within 48 h after delivery	
	Number of biological samples	Number of pregnant women	Average gestational age (Mean, 95%CI/median)	Number of biological samples	Number of pregnant women	Average gestational age (Mean, 95%CI/median)	Number of biological samples	Number of pregnant women	Average gestational age (Mean, 95%CI/median)	Number of biological samples	Number of pregnant women
Serum	25,930	8725	13.5	13,458	4345	24.4	4701	1390	34	846	213
Plasma	25,966	8743	13.5	13,577	4383	24.4	7891	2416	33.4	802	204
Urine	23,368	8687	14.3	10,838	3539	23.6	2257	554	32.4		
Buffy coat	10,288	8602	13.5	5898	4229	24.5	3296	2273	33.4	408	203
CVF				1683	543	28.5	2192	614	34.1		
Amniotic fluid										865	291
Placenta										3321	670
Umbilical cord plasma										1678	663
Umbilical cord buffy coat										819	525
Umbilical cord										707	350
Umbilical cord serum										333	330
Total number of biological samples	85,552			45,454			20,337			9779	

*CVF Cervicovaginal fluid; Total number of biological samples: 161,122

## Discussion

### Unique characteristics of the prospective multicenter birth cohort

In this study, the average age of pregnant women was 29.76 years, with the average age of primiparous women being 28.67 years, slightly higher than the average age of 28.48 years reported in 2015 [38]. The age distribution showed that the highest proportion of pregnant women fell within the 27–30 years age range, followed by the 31–35 years age range, indicating an upward trend in maternal age. Weight gain during pregnancy significantly affects the current and future health of both the mother and the child. With the changing demographic structure of the pregnant population and increasing attention to prenatal nutrition, the number of overweight or obese women post-conception has gradually increased. According to the changes in BMI from pre-pregnancy to delivery in this study, 5.57% of women had a pre-pregnancy BMI of 30 or higher, which increased to 22.16% by the time of delivery. The proportion of women with a pre-pregnancy BMI between 25 and 30 was 18.48%, which nearly tripled to approximately 60% by delivery. This highlights the need for stronger awareness and control of weight gain during pregnancy (Table 2).

In terms of height distribution, 41.08% of the pregnant women were shorter than 160 cm, 34.79% were between 160 and 165 cm, and 24.13% were taller than 165 cm (Table 2). There were differences in average height across provinces (Table 3). For instance, Liaoning Province and Inner Mongolia Autonomous Region, located in northern China, had pregnant women with higher average heights compared to other provinces. Regarding delivery methods, the cesarean section rate was 39.76%, slightly higher than the 36.7% reported in China in 2018 [39]. In addition, pregnant women with a bachelor’s degree accounted for the majority, reaching 68.59% (Table 2), with each province having more than 55% of pregnant women holding a bachelor’s degree, indicating widespread recognition of the importance of education. Additionally, Guangdong province also had the highest proportion of women with two or more children (12.22%), likely due to its developed economy and higher fertility willingness among affluent families.

The prevalence of GDM, preterm birth, PE and FGR observed in this cohort is 16.74%, 4.92%, 2.74%, 1.78% respectively (Table 4), this is consistent with other studies [10, 40–42]. The prevalence of ICP varies between studies [43], and we observed in this cohort was 1.54% (Table 4). ART and advanced maternal age have a strong association with adverse pregnancy outcomes like preterm birth and diabetes [44, 45]. Consistently, we found that compared to women with natural pregnancy, women receiving ART exhibited a significantly higher prevalence of preterm birth, PE, GDM, and PCOS (Supplement Table 1). We also found that compared to women at age 35 and older, women younger than 35 showed a significantly lower prevalence in preterm birth and GDM (Supplement Table 2), which is in accordance with other studies [46]. There is no significant difference between two groups in terms of FGR, PE, ICP, macrosomia, and fetal heart abnormalities. We observed 9 participants with fetal chromosome abnormalities and 4 participants with fetal genetic abnormalities, 13 of whom were conceived naturally and 12 were under 35 years (Supplement Table 3).We postulate that de novo mutation may be the cause, and future research is needed to look deeper into the risk factors of pregnancy complications and fetal chromosome / genetic abnormalities.

### Strengths and weaknesses

This study collected biological samples from pregnant women in 10 provinces of China at various stages of the perinatal period, including serum, plasma, buffy coat, urine, CVF, placenta, amniotic fluid, and umbilical cord blood. Additionally, 161,122 biological samples have been collected in this study (Table 5), the diverse biological samples collected provide a crucial foundation for multi-omics research. Plasma and serum samples can be used to generate omics data, including cell free DNA, cell free RNA, metabolome and proteome data. Buffy coat samples are mainly used to obtain whole genome sequence data. Urine and amniotic fluid samples play an important role in metabolomics and proteomics research. CVF samples can be used to obtain proteomics and metagenomics data. Placental samples can provide whole genome sequences and spatial omics data. Umbilical cord blood samples can obtain whole genome sequences and single-cell omics data. Each sample type has its unique research value and application range, and we have obtained comprehensive information on both the mothers and the newborns, which provides valuable data resources for comprehensive and in-depth maternal and child health research.

The Developmental Origins of Health and Disease (DOHaD) highlights why and how diverse facets of the early life environment and events have long-term effects in shaping health and disease vulnerability in later life [47]. Pregnancy and birth cohorts, particularly prospective, multicenter, multi-omic birth cohorts offer the opportunity to the field of DOHaD, our study provides a comprehensive approach to studying the developmental origins of health and disease, including extensive and diverse data collection, diverse biological samples, longitudinal design and multi-omics integration make it a valuable addition to the existing body of cohort studies. The study has several notable characteristics:

A. The study spans 10 provinces in China, representing diverse geographical environments, climatic conditions, and levels of economic development, which enhances the regional representativeness of the study results. As a multi-ethnic nation, China’s diverse ethnic representation in this study allows the study to reflect the characteristics of different ethnic groups in reproduction health and fetal development. We aim to provide valuable insights into the developmental origins of health and disease, particularly within the context of Chinese populations. The study will contribute to the global understanding of DOHaD by identifying unique and common risk factors across different populations, have the potential to inform public health policies and interventions tailored to improving maternal and child health in China and globally.
B. The study collects a variety of biological samples at all stages of pregnancy, offering unique insights of multi-omics. Serum, Plasma and Urine provide information on circulating cell-free nucleic acids, metabolic and protein profiles, allowing the identification of non-invasive biomarkers. Buffy Coat contains white blood cells, useful for DNA extraction and genetic studies. CVF is important for studying maternal infections and their impact on pregnancy outcomes. Amniotic Fluid provides fetal cells and information on the intrauterine environment. Placenta is crucial for understanding maternal–fetal nutrient exchange and placental function. Umbilical Cord Plasma and Buffy Coat offer insights into the newborn’s immediate health status and genetic background.
C. The study involves a substantial number of pregnant women and comprehensive maternal and child information, and is designed to follow participants from early pregnancy through childhood, providing a large sample size and longitudinal data that are crucial for understanding the developmental origins of health and disease. Detailed information was collected, including demographic characteristics, health status and environmental exposures at various stages of pregnancy. This long-term follow-up allows us to observe how early life exposures impact health outcomes over time.
D. The study offers the integration of multi-omics technologies, including genomics, transcriptomics, metabolomics, and proteomics. Given the high quality requirements of multi-omics testing, we established rigorous quality control standards for the collected biological samples. For example, blood samples had to be processed and separated within four hours of collection to ensure sample integrity and research accuracy. Our multi-omics approach and comprehensive data collection can serve as a model for future cohort studies, providing critical resources for the prevention and scientific investigation of reproductive and developmental diseases. Multi-omics data are currently being generated for following studies.

However, the study also faces certain challenges and limitations. The cohort study spans a lengthy period, covering early pregnancy, mid-pregnancy, late pregnancy, postpartum, newborns, and children stages. This extensive timeframe may result in follow-up losses and sample collection gaps. To address this issue, we have adopted telephone follow-ups to ensure comprehensive collection of pregnancy outcome and neonatal data. While the diversity of the cohort is a strength, it also poses challenges in terms of standardizing data and sample collection across different regions and populations. Efforts and rigorous quality control standards are being made to ensure consistency and comparability of data. Additionally, we are expanding the scope of the study by planning to collect pre-pregnancy samples and paternal samples and information to more fully understand the impact of family and genetic factors on maternal and child health.

### Data access policies

The China Prospective Multi-Center Birth study has an online database that details the information about the participants and their offspring, which is being updated and is currently accessible to project investigators. We are committed to making our data accessible to the broader scientific community, and researchers can obtain access upon request and after ethical and data use approval. We also prioritize the privacy and confidentiality of our participants. All data will be anonymized before sharing to protect participant identities. Interested investigators can contact Zhou Si [zhousi@bgi.com], Zhao Lijian [zhaolijian@genomics.cn] or Qi Hongbo [qihongbo728@163.com].

## Supplementary Information

Below is the link to the electronic supplementary material.

Supplementary file1 (DOCX 146 KB)
