# Supplementary material for: A prospective multicenter birth cohort in China: pregnancy health atlas
Source: Eur J Epidemiol. 2024 Nov 15;39(11):1297–310. doi: 10.1007/s10654-024-01157-x (PMC11646276; doi:10.1007/s10654-024-01157-x)
Supplement: Supplementary file 1 — Supplementary file1 (DOCX 146 KB) [file 10654_2024_1157_MOESM1_ESM.docx]

**Supplement**

**Article title：A prospective multicenter birth cohort in China: Pregnancy Health Atlas**

**Journal name ：European Journal of Epidemiology**

Supplement Fig. 1 Increase of participant number since the launch of the cohort


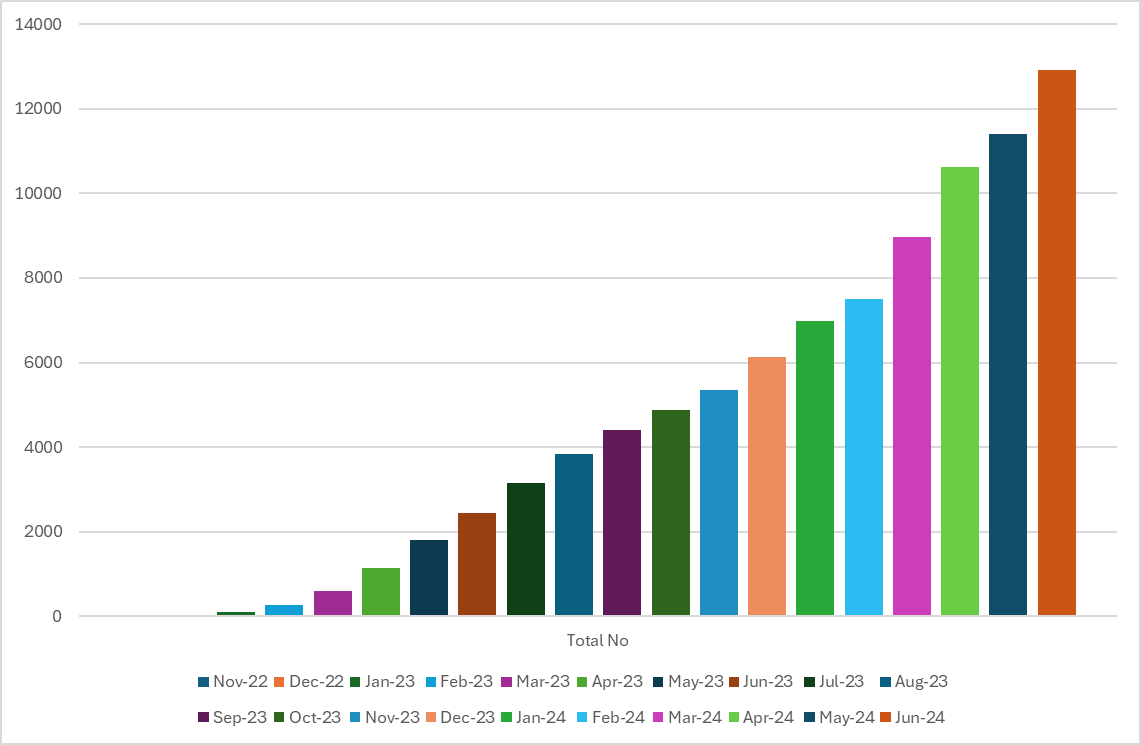


Supplement Fig.2 The proportion of ethnic minorities


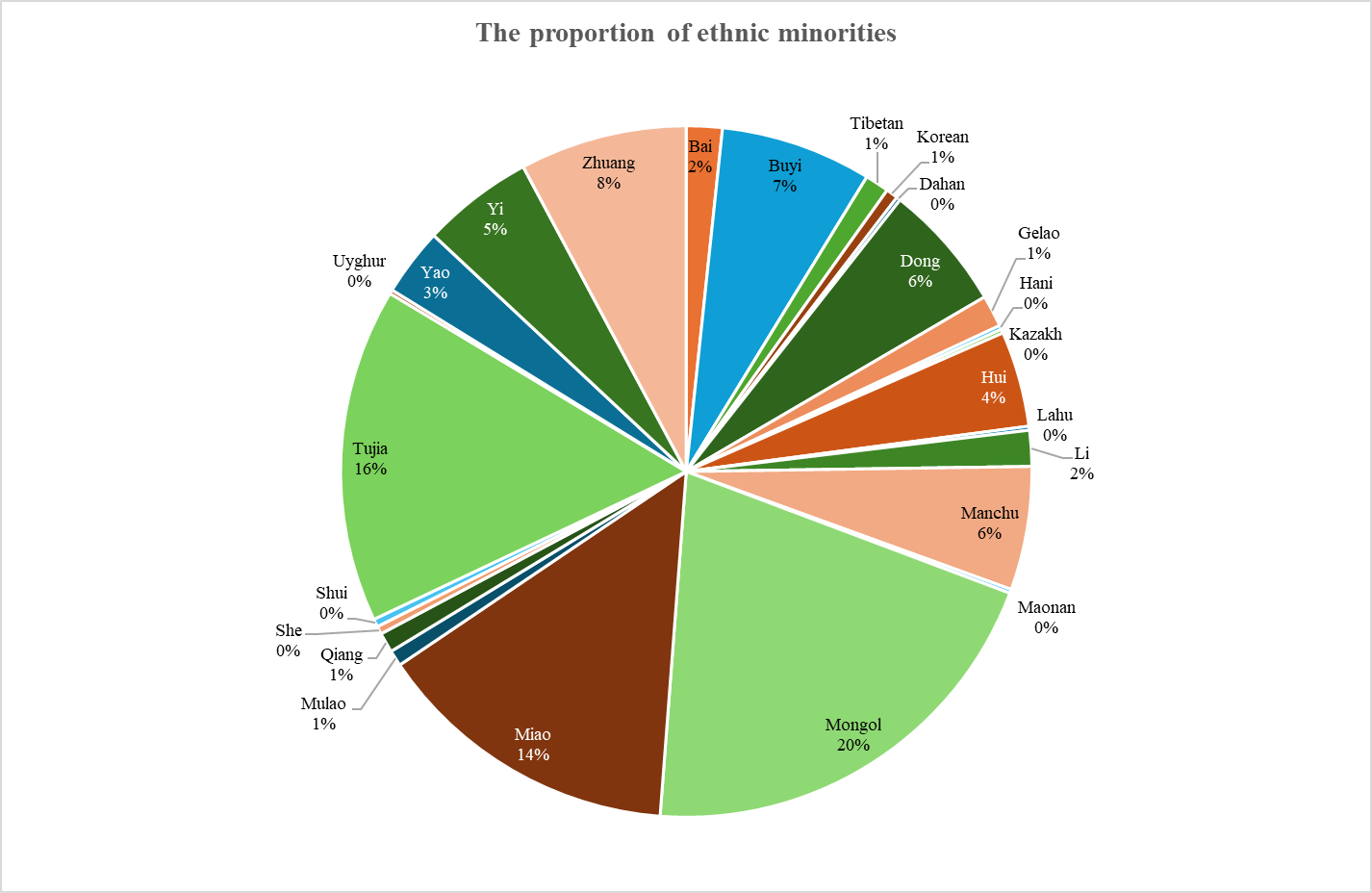


**Supplement Table 1:** Prevalence of pregnancy diseases in two group (classified by modes of conception).

| Disease | ART | | Natural pregnancy | |
| --- | --- | --- | --- | --- |
|  | No. | % | No. | % |
| uterine leiomyoma | 18 | 6.64% | 168 | 3.79% |
| preterm birth | 25 | 9.23% | 227 | 5.12% |
| fetal distress | 12 | 4.43% | 124 | 2.80% |
| Asymptomatic streptococcal infection | 14 | 5.17% | 331 | 7.47% |
| thalassemia | 24 | 8.86% | 342 | 7.72% |
| hypothyroidism | 35 | 12.92% | 375 | 8.47% |
| GDM | 59 | 21.77% | 750 | 16.93% |
| PROM | 47 | 17.34% | 885 | 19.98% |
| PE | 16 | 5.90% | 106 | 2.39% |
| macrosomia | 9 | 3.32% | 152 | 3.43% |
| PCOS | 5 | 1.85% | 30 | 0.68% |

* GDM: gestational diabetes mellitus; PCOS: polycystic ovary syndrome, PE: preeclampsia; PROM: premature rupture of membranes

**Supplement Table 2:** Prevalence of pregnancy diseases in two group (classified by maternal age).

| Disease | 34 or younger | | 35 or older | |
| --- | --- | --- | --- | --- |
|  | No. | % | No. | % |
| preterm birth | 220 | 5.27% | 56 | 8.15% |
| FGR | 84 | 2.01% | 11 | 1.60% |
| GDM | 615 | 14.74% | 212 | 30.86% |
| gestational hypertension | 118 | 2.83% | 24 | 3.49% |
| PE | 100 | 2.40% | 23 | 3.35% |
| ICP | 12 | 0.29% | 2 | 0.29% |

* ICP: intrahepatic cholestasis of pregnancy; FGR: fetal growth restriction; GDM: gestational diabetes mellitus; PE: preeclampsia;

**Supplement Table 3**: Identified individuals with chromosome / genetic abnormalities

|  | Age | Mode of conception |
| --- | --- | --- |
| No.1 | 38 | natural pregnancy |
| No.2 | 24 | natural pregnancy |
| No.3 | 19 | natural pregnancy |
| No.4 | 28 | natural pregnancy |
| No.5 | 28 | natural pregnancy |
| No.6 | 31 | natural pregnancy |
| No.7 | 34 | natural pregnancy |
| No.8 | 28 | natural pregnancy |
| No.9 | 30 | natural pregnancy |
| No.10 | 30 | natural pregnancy |
| No.11 | 32 | natural pregnancy |
| No.12 | 29 | natural pregnancy |
| No.13 | 27 | natural pregnancy |
